# Supplementary material for: Remodeling of lumbar motor circuitry remote to a thoracic spinal cord injury promotes locomotor recovery
Source: eLife. 2018 Sep 12;7:e39016. doi: 10.7554/eLife.39016 (PMC6170189; doi:10.7554/eLife.39016)
Supplement: Transparent reporting form [file elife-39016-transrepform.docx]

***eLife’s* transparent reporting form**

We encourage authors to provide detailed information *within their submission* to facilitate the interpretation and replication of experiments. Authors can upload supporting documentation to indicate the use of appropriate reporting guidelines for health-related research (see [EQUATOR Network](http://www.equator-network.org/%20)), life science research (see the [BioSharing Information Resource](https://biosharing.org/)), or the [ARRIVE guidelines](http://www.plosbiology.org/article/info:doi/10.1371/journal.pbio.1000412) for reporting work involving animal research. Where applicable, authors should refer to any relevant reporting standards documents in this form.

If you have any questions, please consult our Journal Policies and/or contact us: [editorial@elifesciences.org](mailto:editorial@elifesciences.org).

**Sample-size estimation**

- You should state whether an appropriate sample size was computed when the study was being designed
- You should state the statistical method of sample size computation and any required assumptions
- If no explicit power analysis was used, you should describe how you decided what sample (replicate) size (number) to use

Please outline where this information can be found within the submission (e.g., sections or figure legends), or explain why this information doesn’t apply to your submission:

For this study, a power analysis has been conducted based on the previous and pilot studies to determine the appropriate sample sizes. The test is under the assumption that at least 1 experimental group has an effect size of 1.5 SD when compared to the control group. All reported groups were above the minimum calculated sample size. The detailed information of sample sizes for each animal group can be found on Table 1 & 2.

**Replicates**

- You should report how often each experiment was performed
- You should include a definition of biological versus technical replication
- The data obtained should be provided and sufficient information should be provided to indicate the number of independent biological and/or technical replicates
- If you encountered any outliers, you should describe how these were handled
- Criteria for exclusion/inclusion of data should be clearly stated
- High-throughput sequence data should be uploaded before submission, with a private link for reviewers provided (these are available from both GEO and ArrayExpress)

Please outline where this information can be found within the submission (e.g., sections or figure legends), or explain why this information doesn’t apply to your submission:

The **biological replication** we defined in our study is that the parallel measurements of animal samples which capture random biological variation. The **technical replication** is the repetitive measurements during the same experiments. In our case, an example would be 3 repetitions have been done for NT-3 ELISA *in vitro* assay in figure 1G.

For detailed information, a table has been included, please see **Data Checklist_WY.**

If a value is outside of the range of 3 times of SD ± the average value, it would be considered as an outlier to be excluded.

**Statistical reporting**

- Statistical analysis methods should be described and justified
- Raw data should be presented in figures whenever informative to do so (typically when N per group is less than 10)
- For each experiment, you should identify the statistical tests used, exact values of N, definitions of center, methods of multiple test correction, and dispersion and precision measures (e.g., mean, median, SD, SEM, confidence intervals; and, for the major substantive results, a measure of effect size (e.g., Pearson's r, Cohen's d)
- Report exact p-values wherever possible alongside the summary statistics and 95% confidence intervals. These should be reported for all key questions and not only when the p-value is less than 0.05.

Please outline where this information can be found within the submission (e.g., sections or figure legends), or explain why this information doesn’t apply to your submission:

In general, all statistical analysis was performed by two-tailed Student’s t tests, one-way ANOVA, and Tukey’s post-hoc test between all groups in each individual experiment. The early response rate of tcMMEP was performed with χ2 test and BMS analysis was performed with repeated measures two-way ANOVA. Regression analysis was performed in behavioral and dendrite length examinations for each animal. Regression values (r) were identified by comparing two individual variables. The Pearson correlation coefficients were identified for parametric data sets. For details, we have an excel file including a summary excel table of statistical report on all figures (see **Data Checklist_WY**)

(For large datasets, or papers with a very large number of statistical tests, you may upload a single table file with tests, Ns, etc., with reference to sections in the manuscript.)

**Group allocation**

- Indicate how samples were allocated into experimental groups (in the case of clinical studies, please specify allocation to treatment method); if randomization was used, please also state if restricted randomization was applied
- Indicate if masking was used during group allocation, data collection and/or data analysis

Please outline where this information can be found within the submission (e.g., sections or figure legends), or explain why this information doesn’t apply to your submission:

For allocation of animal groups, we used randomization to assign animals to each group throughout this study. For randomization, we ensured equal number of animals for each group and the ratio of female and male animals is 1:1. During group allocation, data collection, and data analysis, the testers were double-blinded.

**Additional data files (“source data”)**

- We encourage you to upload relevant additional data files, such as numerical data that are represented as a graph in a figure, or as a summary table
- Where provided, these should be in the most useful format, and they can be uploaded as “Source data” files linked to a main figure or table
- Include model definition files including the full list of parameters used
- Include code used for data analysis (e.g., R, MatLab)
- Avoid stating that data files are “available upon request”

Please indicate the figures or tables for which source data files have been provided:

An excel file has been provided (**Data Checklist_WY.)** to showcase a summary of all statistical reports.
